# Crystal structure of 2-oxo-2*H*-chromen-3-yl propano­ate

**DOI:** 10.1107/S2056989016015279

**Published:** 2016-10-11

**Authors:** Eric Ziki, Jules Yoda, Abdoulaye Djandé, Adama Saba, Rita Kakou-Yao

**Affiliations:** a Laboratoire de Cristallographie et Physique Moléculaire, UFR SSMT, Université Félix Houphouët Boigny de Cocody 22 BP 582 Abidjan 22, Côte d’Ivoire; b Laboratoire de Chimie Moléculaire et Matériaux, Equipe de Chimie Organique et Phytochimie, Université Ouaga I Pr Joseph KI-ZERBO 03 BP 7021 Ouagadougou 03, Burkina Faso

**Keywords:** crystal structure, π–π inter­actions, C—H⋯π inter­actions, chromane, quantum-chemical calculations

## Abstract

In the title compound, C_12_H_10_O_4_, the dihedral angle between the coumarin ring system [maximum deviation = 0.033 (8) Å] and the propionate side chain is 78.48 (8)°. In the crystal, weak C—H⋯O hydrogen bonds generate inversion dimers and and C—H⋯π and π–π inter­actions link the dimers into a three-dimensional network. A quantum chemical calculation is in good agreement with the observed structure.

## Abstract 2

In the title compound, C_12_H_9_O_4_, the dihedral angle between the coumarin ring system and the propionate side chain is 78.48 (8)°.

## Chemical context   

Coumarin and its derivatives are widely recognized for their multiple biological activities, including anti­cancer (Lacy *et al.*, 2004 ▸; Kostova, 2005 ▸), anti-inflammatory (Todeschini *et al.*, 1998 ▸), anti­viral (Borges *et al.*, 2005 ▸), anti­malarial (Agarwal *et al.*, 2005 ▸) and anti­coagulant (Maurer *et al.*, 1998 ▸) properties. As part of our studies in this area, we now describe the synthesis and crystal structure of the title compound, (I).
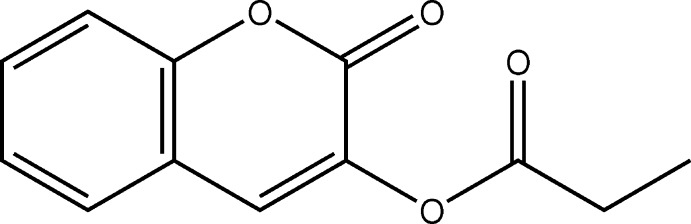


## Structural commentary   

**Figure 1:**
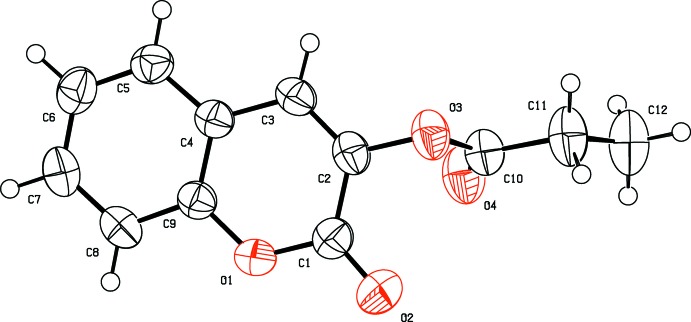
The mol­ecular structure of compound (I), with displacement ellipsoids drawn at the 50% probability level.

In compound (I) (Fig. 1 ▸), the coumarin ring system is almost planar [maximum deviation = 0.033 (1)Å] and is oriented at an angle of 70.84 (8)° with respect to the plane formed by the propano­ate group. An inspection of the bond lengths shows that there is a slight asymmetry of the electronic distribution around the coumarin ring: the C2—C3 [1.329 (2) Å] and C2—C1 [1.460 (2) Å] bond lengths are shorter and longer, respectively, than those expected for a C_ar_—C_ar_ bond. This suggests that the electron density is preferentially located in the C2—C3 bond at the pyrone ring, as seen in other coumarin-3-carboxamide derivatives (Gomes *et al.*, 2016 ▸).

## Supra­molecular features   

**Figure 2:**
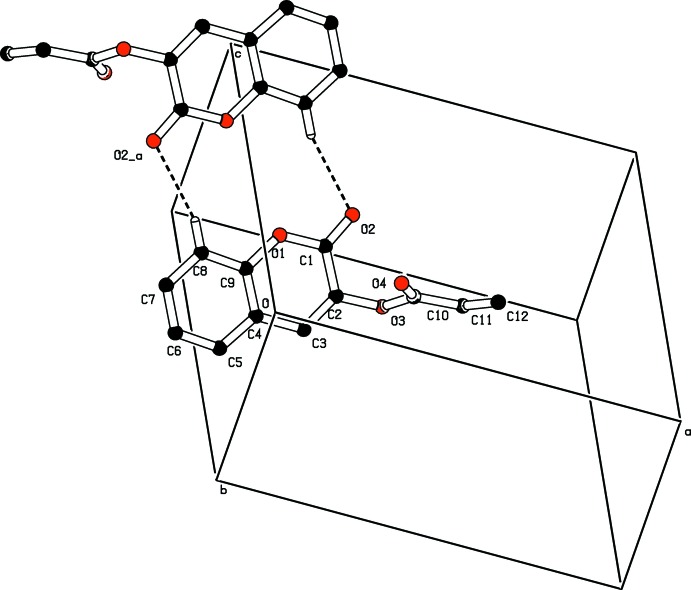
View of an inversion dimer linked by a pair of C8—H8⋯O2 (−*x*, −*y*, −*z* + 1) inter­actions, generating an 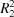
(12) loop. This dimers stack by unit translation along the *c* axis. H atoms not involved in hydrogen bonding have been omitted.

**Figure 3:**
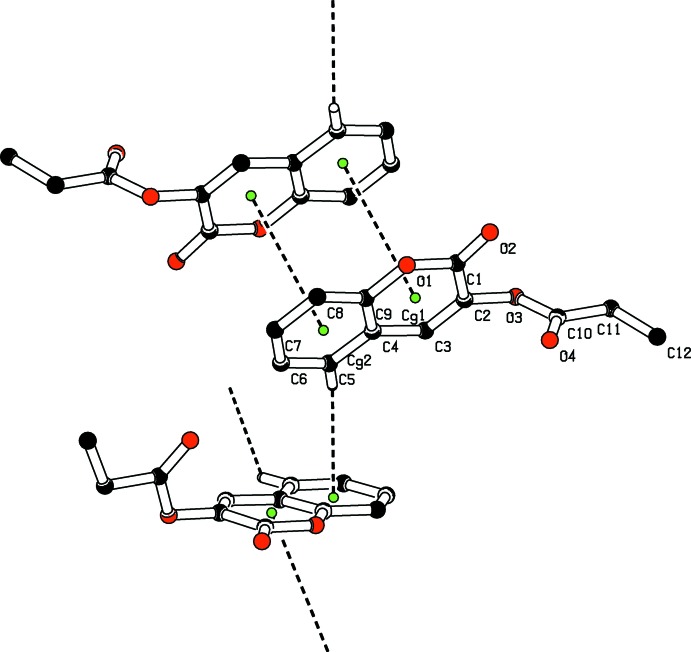
A view of the crystal packing, showing the π–π stacking and C—H⋯π inter­actions (dashed lines). The green dots are ring centroids. H atoms not involved in the C—H⋯π inter­actions have been omitted for clarity.

In the crystal, the mol­ecules are linked by pairs of C8—H8⋯O2(*x*, −*y*, 1 − *z*) weak hydrogen bonds to form 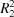
(12) loops, which lie in a chain running along the *c* axis direction (Fig. 2 ▸). Weak aromatic π–π stacking inter­actions of 3.7956 (8) Å (Janiak, 2000 ▸) are present between the coumarin pyran ring (centroid *Cg*1) and benzene ring (centroid *Cg*2) of symmetry-related (−*x*, 1 − *y*, 1 − *z*) mol­ecules, thus forming a three-dimensional supra­molecular network. A weak C—H⋯*Cg* (π–ring) inter­action is also present (Figs. 3 ▸ and 4 ▸, and Table 1 ▸).

## Theoretical calculations   

Quantum-chemical calculations were performed to compare with the experimental analysis. An *ab-initio* Hartree–Fock (HF) method was used with the standard basis set of 6-31G using the *GAUSSIAN03* software package (Frisch *et al.*, 2004 ▸; Dennington *et al.*, 2007 ▸) to obtain the optimized mol­ecular structure. The computational results are in good agreement with the experimental crystallographic data (Table 2 ▸).

## Synthesis and crystallization   

In a 100 ml round-necked flask topped with a water condenser were introduced successively 25 ml of dried diethyl ether, 6.17 × 10 ^−3^ mol (≃ 0.8 ml) of propionic anhydride and 2.35 ml (4.7 molar equivalents) of dried pyridine. While stirring strongly, 6.17 × 10^−3^ mol (1 g) of 3-hy­droxy­coumarin was added in small portions over 30 min. The reaction mixture was left under agitation at room temperature for 3 h. The mixture was then poured in a separating funnel containing 40 ml of chloro­form and washed with diluted hydro­chloric acid solution until the pH was 2–3. The organic layer was extracted, washed with water to neutrality, dried over MgSO_4_ and the solvent removed. The resulting precipitate (crude product) was filtered off with petroleum ether and recrystallized from a solvent mixture of chloro­form–hexane (1/3, *v*/*v*). Colourless prisms of the title compound were obtained in a yield of 65%, m. p. = 351–353 K.

## Refinement   

Crystal data, data collection and structure refinement details are summarized in Table 3 ▸. H atoms were placed in calculated positions [C—H = 0.93 (aromatic), 0.96 (meth­yl) or 0.97 Å (methyl­ene)] and refined using a riding-model approximation with *U*_iso_(H) constrained to 1.2 (aromatic and methyl­ene group) or 1.5 (methyl group) times *U*_eq_ of the respective parent atom.

## Supplementary Material

Crystal structure: contains datablock(s) I, New_Global_Publ_Block. DOI: 10.1107/S2056989016015279/hb7613sup1.cif

Structure factors: contains datablock(s) I. DOI: 10.1107/S2056989016015279/hb7613Isup2.hkl

Supporting information file. DOI: 10.1107/S2056989016015279/hb7613Isup3.cml

CCDC reference: 1507161

Additional supporting information: crystallographic information; 3D view; checkCIF report

## Figures and Tables

**Figure 4:**
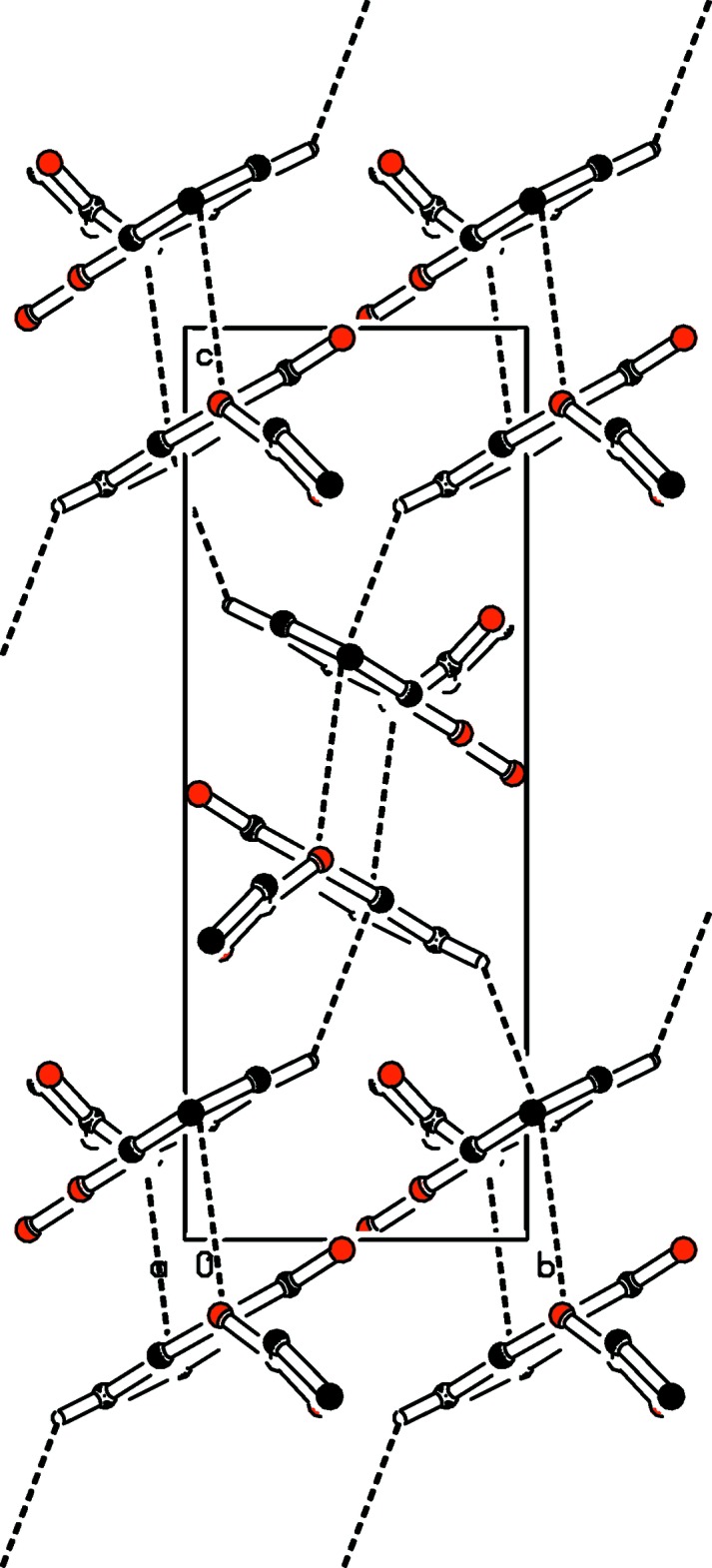
Part of the crystal structure of (I), showing C—H⋯π and π–π inter­actions as dashed lines. H atoms have been omitted for clarity.

**Table 1:** Hydrogen-bond geometry (Å, °) *Cg*2 is the centroid of the C4–C9 ring.

*D*—H⋯*A*	*D*—H	H⋯*A*	*D*⋯*A*	*D*—H⋯*A*
C8—H8⋯O2^i^	0.93	2.59	3.4783 (19)	161
C5—H5⋯*Cg*2^ii^	0.93	2.78	3.4959 (16)	134

Symmetry codes: (i) 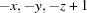; (ii) 
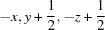
.

**Table 2:** Experimental and calculated bond lengths (Å)

Bond	X-ray	HF(6–31G)
O1—C1	1.3628 (17)	1.371
O1—C9	1.3769 (17)	1.378
O2—C1	1.2004 (18)	1.227
O3—C10	1.3713 (18)	1.359
O3—C2	1.3893 (17)	1.381
O4—C10	1.1932 (19)	1.21
C1—C2	1.460 (2)	1.468
C2—C3	1.329 (2)	1.355
C3—C4	1.4403 (19)	1.441
C4—C5	1.401 (2)	1.406
C4—C9	1.3928 (18)	1.407
C5—C6	1.370 (2)	1.387
C6—C7	1.386 (2)	1.395
C7—C8	1.379 (2)	1.383
C8—C9	1.3842 (19)	1.408
C10—C11	1.495 (2)	1.497
C11—C12	1.491 (3)	1.525

**Table 3:** Experimental details

Crystal data	
Chemical formula	C_12_H_10_O_4_
*M*_r_	218.20
Crystal system, space group	Monoclinic, *P*2_1_/*c*
Temperature (K)	293
*a*, *b*, *c* (Å)	12.1179 (4), 5.7243 (2), 15.3275 (5)
β (°)	94.881 (3)
*V* (Å^3^)	1059.36 (6)
*Z*	4
Radiation type	Cu *K*α
μ (mm^−1^)	0.87
Crystal size (mm)	0.46 × 0.16 × 0.08

Data collection	
Diffractometer	Agilent SuperNova Dual (Cu at zero) Source diffractometer with an AtlasS2 detector
Absorption correction	Multi-scan (*CrysAlis PRO*; Agilent, 2014 ▸)
*T*_min_, *T*_max_	0.778, 1.000
No. of measured, independent and observed [*I* > 2σ(*I*)] reflections	6028, 1930, 1655
*R*_int_	0.020
(sin θ/λ)_max_ (Å^−1^)	0.605

Refinement	
*R*[*F* ^2^ > 2σ(*F* ^2^)], *wR*(*F* ^2^), *S*	0.038, 0.117, 1.06
No. of reflections	1930
No. of parameters	145
H-atom treatment	H-atom parameters constrained
Δρ_max_, Δρ_min_ (e Å^−3^)	0.16, −0.16

Computer programs: *CrysAlis PRO* (Agilent, 2014 ▸), *SHELXS97* (Sheldrick, 2008 ▸), *SHELXL2013* (Sheldrick, 2015 ▸), *PLATON* (Spek, 2009 ▸) and *publCIF* (Westrip, 2010 ▸).

## supplementary crystallographic information

### Crystal data

**Table:**

C_12_H_10_O_4_	*F*(000) = 456
*M**_r_* = 218.20	*D*_x_ = 1.368 Mg m^−^^3^
Monoclinic, *P*2_1_/*c*	Melting point: 351 K
Hall symbol: -P 2ybc	Cu *K*α radiation, λ = 1.54184 Å
*a* = 12.1179 (4) Å	Cell parameters from 3028 reflections
*b* = 5.7243 (2) Å	θ = 5.8–68.6°
*c* = 15.3275 (5) Å	µ = 0.87 mm^−^^1^
β = 94.881 (3)°	*T* = 293 K
*V* = 1059.36 (6) Å^3^	Prism, colourless
*Z* = 4	0.46 × 0.16 × 0.08 mm

### Data collection

**Table:**

Agilent SuperNova Dual (Cu at zero) Source diffractometer with an AtlasS2 detector	1930 independent reflections
Radiation source: sealed X-ray tube	1655 reflections with *I* > 2σ(*I*)
Mirror monochromator	*R*_int_ = 0.020
Detector resolution: 5.3048 pixels mm^-1^	θ_max_ = 68.9°, θ_min_ = 3.7°
ω scan	*h* = −14→14
Absorption correction: multi-scan (CrysAlis PRO; Agilent, 2014)	*k* = −6→6
*T*_min_ = 0.778, *T*_max_ = 1.000	*l* = −15→18
6028 measured reflections	

### Refinement

**Table:**

Refinement on *F*^2^	Primary atom site location: structure-invariant direct methods
Least-squares matrix: full	Secondary atom site location: difference Fourier map
*R*[*F*^2^ > 2σ(*F*^2^)] = 0.038	Hydrogen site location: inferred from neighbouring sites
*wR*(*F*^2^) = 0.117	H-atom parameters constrained
*S* = 1.06	*w* = 1/[σ^2^(*F*_o_^2^) + (0.0631*P*)^2^ + 0.1085*P*] where *P* = (*F*_o_^2^ + 2*F*_c_^2^)/3
1930 reflections	(Δ/σ)_max_ < 0.001
145 parameters	Δρ_max_ = 0.16 e Å^−^^3^
0 restraints	Δρ_min_ = −0.16 e Å^−^^3^
40 constraints	

### Special details

**Table:**

Geometry. All esds (except the esd in the dihedral angle between two l.s. planes) are estimated using the full covariance matrix. The cell esds are taken into account individually in the estimation of esds in distances, angles and torsion angles; correlations between esds in cell parameters are only used when they are defined by crystal symmetry. An approximate (isotropic) treatment of cell esds is used for estimating esds involving l.s. planes.

### Fractional atomic coordinates and isotropic or equivalent isotropic displacement parameters (Å^2^)

**Table:**

	*x*	*y*	*z*	*U*_iso_*/*U*_eq_	
O1	0.08845 (8)	0.19504 (17)	0.44462 (6)	0.0505 (3)	
O3	0.36752 (8)	0.39585 (19)	0.41680 (7)	0.0567 (3)	
C9	0.02550 (11)	0.3708 (2)	0.40435 (8)	0.0426 (3)	
C2	0.25245 (11)	0.3964 (2)	0.40998 (9)	0.0466 (3)	
C3	0.19407 (11)	0.5719 (2)	0.37284 (8)	0.0455 (3)	
H3	0.2298	0.6985	0.3497	0.055*	
C4	0.07494 (11)	0.5644 (2)	0.36879 (8)	0.0420 (3)	
C8	−0.08834 (12)	0.3450 (3)	0.40123 (9)	0.0521 (3)	
H8	−0.1198	0.2150	0.4258	0.063*	
C1	0.20130 (12)	0.1982 (2)	0.45088 (9)	0.0492 (3)	
O4	0.36176 (9)	0.1032 (2)	0.31915 (8)	0.0683 (3)	
O2	0.24985 (10)	0.0406 (2)	0.48893 (8)	0.0695 (3)	
C5	0.00553 (12)	0.7361 (2)	0.32863 (9)	0.0497 (3)	
H5	0.0362	0.8676	0.3045	0.060*	
C6	−0.10727 (13)	0.7114 (3)	0.32463 (10)	0.0566 (4)	
H6	−0.1527	0.8257	0.2975	0.068*	
C7	−0.15398 (12)	0.5169 (3)	0.36080 (10)	0.0567 (4)	
H7	−0.2306	0.5022	0.3578	0.068*	
C10	0.41542 (12)	0.2272 (3)	0.36892 (10)	0.0530 (3)	
C11	0.53858 (13)	0.2315 (4)	0.38698 (13)	0.0728 (5)	
H11A	0.5644	0.3905	0.3808	0.087*	
H11B	0.5576	0.1839	0.4471	0.087*	
C12	0.59736 (16)	0.0768 (5)	0.32791 (15)	0.0858 (6)	
H12A	0.6758	0.0871	0.3428	0.129*	
H12B	0.5806	0.1252	0.2683	0.129*	
H12C	0.5736	−0.0817	0.3346	0.129*	

### Atomic displacement parameters (Å^2^)

**Table:**

	*U*^11^	*U*^22^	*U*^33^	*U*^12^	*U*^13^	*U*^23^
O1	0.0500 (5)	0.0463 (5)	0.0557 (5)	−0.0037 (4)	0.0068 (4)	0.0091 (4)
O3	0.0398 (5)	0.0614 (6)	0.0686 (6)	−0.0025 (4)	0.0019 (4)	−0.0145 (5)
C9	0.0453 (7)	0.0438 (6)	0.0394 (6)	−0.0015 (5)	0.0069 (5)	−0.0014 (5)
C2	0.0395 (7)	0.0505 (7)	0.0499 (7)	−0.0036 (5)	0.0052 (5)	−0.0083 (5)
C3	0.0468 (7)	0.0425 (7)	0.0485 (7)	−0.0072 (5)	0.0109 (5)	−0.0037 (5)
C4	0.0459 (7)	0.0411 (6)	0.0398 (6)	−0.0018 (5)	0.0083 (5)	−0.0037 (5)
C8	0.0473 (7)	0.0570 (8)	0.0535 (7)	−0.0083 (6)	0.0125 (6)	−0.0022 (6)
C1	0.0506 (7)	0.0490 (7)	0.0479 (7)	0.0031 (6)	0.0034 (5)	0.0009 (6)
O4	0.0510 (6)	0.0805 (8)	0.0730 (7)	−0.0006 (5)	0.0025 (5)	−0.0238 (6)
O2	0.0665 (7)	0.0669 (7)	0.0749 (7)	0.0122 (6)	0.0041 (6)	0.0197 (6)
C5	0.0575 (8)	0.0450 (7)	0.0474 (7)	0.0025 (6)	0.0100 (6)	0.0013 (5)
C6	0.0548 (8)	0.0611 (9)	0.0540 (7)	0.0145 (7)	0.0065 (6)	−0.0010 (6)
C7	0.0411 (7)	0.0715 (9)	0.0584 (8)	0.0027 (6)	0.0097 (6)	−0.0069 (7)
C10	0.0450 (8)	0.0594 (8)	0.0545 (7)	0.0007 (6)	0.0038 (6)	−0.0053 (6)
C11	0.0430 (8)	0.0917 (13)	0.0829 (11)	0.0053 (8)	−0.0003 (7)	−0.0168 (10)
C12	0.0526 (10)	0.1094 (16)	0.0951 (13)	0.0179 (10)	0.0050 (9)	−0.0168 (12)

### Geometric parameters (Å, º)

**Table:**

O1—C1	1.3628 (17)	O4—C10	1.1932 (19)
O1—C9	1.3769 (17)	C5—C6	1.370 (2)
O3—C10	1.3713 (18)	C5—H5	0.9300
O3—C2	1.3893 (17)	C6—C7	1.386 (2)
C9—C8	1.3842 (19)	C6—H6	0.9300
C9—C4	1.3926 (18)	C7—H7	0.9300
C2—C3	1.329 (2)	C10—C11	1.495 (2)
C2—C1	1.460 (2)	C11—C12	1.491 (3)
C3—C4	1.4403 (19)	C11—H11A	0.9700
C3—H3	0.9300	C11—H11B	0.9700
C4—C5	1.401 (2)	C12—H12A	0.9600
C8—C7	1.379 (2)	C12—H12B	0.9600
C8—H8	0.9300	C12—H12C	0.9600
C1—O2	1.2004 (18)		
			
C1—O1—C9	122.43 (10)	C4—C5—H5	119.8
C10—O3—C2	115.41 (11)	C5—C6—C7	120.31 (14)
O1—C9—C8	116.74 (12)	C5—C6—H6	119.8
O1—C9—C4	121.11 (12)	C7—C6—H6	119.8
C8—C9—C4	122.15 (13)	C8—C7—C6	120.90 (14)
C3—C2—O3	121.88 (12)	C8—C7—H7	119.6
C3—C2—C1	122.80 (12)	C6—C7—H7	119.6
O3—C2—C1	115.22 (12)	O4—C10—O3	121.89 (13)
C2—C3—C4	119.40 (12)	O4—C10—C11	127.56 (15)
C2—C3—H3	120.3	O3—C10—C11	110.52 (13)
C4—C3—H3	120.3	C12—C11—C10	113.50 (15)
C9—C4—C5	117.88 (12)	C12—C11—H11A	108.9
C9—C4—C3	118.03 (12)	C10—C11—H11A	108.9
C5—C4—C3	124.05 (12)	C12—C11—H11B	108.9
C7—C8—C9	118.32 (13)	C10—C11—H11B	108.9
C7—C8—H8	120.8	H11A—C11—H11B	107.7
C9—C8—H8	120.8	C11—C12—H12A	109.5
O2—C1—O1	118.14 (13)	C11—C12—H12B	109.5
O2—C1—C2	125.74 (14)	H12A—C12—H12B	109.5
O1—C1—C2	116.12 (12)	C11—C12—H12C	109.5
C6—C5—C4	120.43 (13)	H12A—C12—H12C	109.5
C6—C5—H5	119.8	H12B—C12—H12C	109.5
			
C1—O1—C9—C8	179.07 (12)	C9—O1—C1—C2	−1.73 (18)
C1—O1—C9—C4	−0.99 (18)	C3—C2—C1—O2	−176.26 (14)
C10—O3—C2—C3	−113.91 (15)	O3—C2—C1—O2	0.3 (2)
C10—O3—C2—C1	69.53 (17)	C3—C2—C1—O1	3.69 (19)
O3—C2—C3—C4	−179.09 (11)	O3—C2—C1—O1	−179.78 (10)
C1—C2—C3—C4	−2.8 (2)	C9—C4—C5—C6	−0.27 (19)
O1—C9—C4—C5	179.85 (11)	C3—C4—C5—C6	177.46 (12)
C8—C9—C4—C5	−0.21 (19)	C4—C5—C6—C7	0.4 (2)
O1—C9—C4—C3	1.98 (17)	C9—C8—C7—C6	−0.4 (2)
C8—C9—C4—C3	−178.08 (12)	C5—C6—C7—C8	−0.1 (2)
C2—C3—C4—C9	−0.08 (18)	C2—O3—C10—O4	6.2 (2)
C2—C3—C4—C5	−177.81 (12)	C2—O3—C10—C11	−175.47 (14)
O1—C9—C8—C7	−179.53 (12)	O4—C10—C11—C12	6.5 (3)
C4—C9—C8—C7	0.5 (2)	O3—C10—C11—C12	−171.65 (17)
C9—O1—C1—O2	178.23 (13)		

### Hydrogen-bond geometry (Å, º)

Cg2 is the centroid of the C4–C9 ring.

**Table:**

*D*—H···*A*	*D*—H	H···*A*	*D*···*A*	*D*—H···*A*
C8—H8···O2^i^	0.93	2.59	3.4783 (19)	161
C5—H5···*Cg*2^ii^	0.93	2.78	3.4959 (16)	134

Symmetry codes: (i) −*x*, −*y*, −*z*+1; (ii) −*x*, *y*+1/2, −*z*+1/2.
